# Relationship Among Global Femoral Offset, Leg Lengthening, and Tibiofemoral Rotation After Total Hip Arthroplasty

**DOI:** 10.3390/jcm14092893

**Published:** 2025-04-23

**Authors:** Norio Imai, Yuki Hirano, Daisuke Homma, Yuki Komuta, Yoji Horigome, Hiroyuki Kawashima

**Affiliations:** 1 Division of Comprehensive Musculoskeletal Medicine, Niigata University Graduate School of Medical and Dental Sciences, 1-757, Asahimachi-dori, Chuo-ward, Niigata City 951-8510, Niigata Prefecture, Japan; 2 Division of Orthopedic Surgery, Department of Regenerative and Transplant Medicine, Niigata University Graduate School of Medical and Dental Sciences, 1-757, Asahimachi-dori, Chuo-ward, Niigata City 951-8510, Niigata Prefecture, Japan

**Keywords:** total hip arthroplasty, global femoral offset, tibiofemoral, rotation, patella tilt, leg lengthening

## Abstract

**Background/Objectives**: Several studies have described the changes in tibiofemoral rotation (TFRA) and patellar tilt angle (PTA) following total hip arthroplasty (THA). However, no studies have applied three-dimensional measurements to evaluate leg lengthening, changes in global femoral offset (GFO) or TFRA, or PTA during THA. Accordingly, this study employs three-dimensional measurements to test our hypothesis that increased leg lengthening is associated with an increase in TFRA, which increases PTA and worsens postoperative m-Harris Hip Score (mHHS). **Methods**: A total of 111 consecutive patients who underwent THA were enrolled. THA-related changes in GFO, femoral version, TFRA, PTA, and leg lengthening. Relationships between each parameter and the m-Harris Hip Sc were also assessed using the intraclass correlation coefficient. **Results:** Leg lengthening was significantly positively correlated with changes in TFRA and PTA. However, changes in GFO negatively correlated with changes in TFRA and PTA. Moreover, changes in GFO and leg lengthening were the only factors affecting changes in TFRA and PTA, respectively. **Conclusions**: Direct relationships exist between changes in GFO and changes in TFRA and PTA. This may be related to increased tension of the adductor muscles and medial soft tissue around the knee, ultimately reducing strain on the patellofemoral joint and improving knee pain.

## 1. Introduction

Total hip arthroplasty (THA) reduces motion and rest pain and enhances hip function during walking and daily living activities in patients with hip osteoarthritis (HOA) and osteonecrosis of the femoral head [1,2,3,4,5]. Over 90% of patients who undergo THA survive for at least 20 years post-surgery [6], with survivorship of 30 years or more expected. Indeed, the number of THAs performed annually continues to increase [7], with an estimated 5% annual increase in THAs performed in the United States since 2000, which is predicted to reach 635,000 surgeries in 2030 [8].

**Figure 1:**
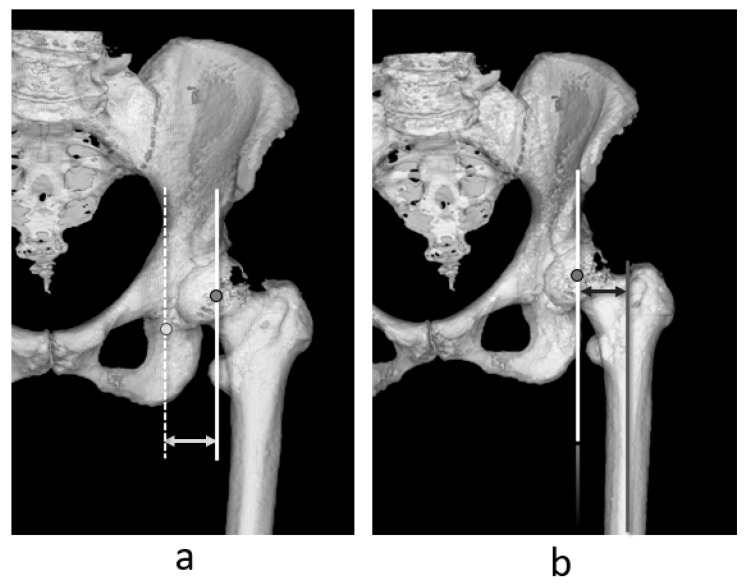
Definition of acetabular offset (**a**) and femoral offsets (**b**).

Recent reports have indicated that when the sum of the leg length discrepancy and the difference in the global femoral offset (GFO), acetabular offset (AO), and femoral offset (FO) (Figure 1) equals that of the intact healthy side, postoperative outcomes are improved [9,10,11,12].

Several studies have described the changes in tibiofemoral rotation and patellar tilt following THA. However, while Yu et al. reported that the tibia is externally rotated after THA [13], the underlying cause(s) have not been defined. Meanwhile, Tokuhara et al. [14] reported that the extent of leg lengthening during THA is proportional to the increase in patellar tilt and external rotation of the patella relative to the femur. Specifically, leg lengthening of ≥17 mm causes anterior knee pain. Furthermore, we previously reported that the tibia becomes externally rotated by approximately 10° and the patella by 7° in developmental dysplasia of the hip (DDH) cases compared with healthy cases [15,16,17]. Nevertheless, no reports have employed three-dimensional (3D) measurements to assess leg lengthening, GFO changes, and changes in the tibiofemoral rotation angle (TFRA) and patellar tilt angle (PTA) during THA.

We hypothesize that an increase in leg lengthening is associated with an increase in TFRA, which increases PTA and worsens the postoperative m-Harris Hip Score (mHHS), whereas GFO does not contribute to TFRA or PTA changes. To test this hypothesis, the current study uses 3D measurements to investigate the relationships between changes in leg lengthening and GFO during THA and changes in TFRA and PTA.

## 2. Materials and Methods

### 2.1. Study Design

This “analytical study” was retrospective and cross-sectional. Computed tomography (CT) images captured before and after THA and medical records were evaluated.

### 2.2. Participants

Patients who underwent THA at our institution between 1 June 2012, and 30 April, 2021, were evaluated. A total of 273 THAs were performed during the study period. The inclusion criteria were as follows: (1) hemilateral HOA, with no pain or symptoms on the non-surgical healthy side; (2) a center-edge angle < 25° on the healthy side in plain anteroposterior radiograph.

Exclusion criteria included (1) previous surgery on the hip joint of the THA side; (2) symptoms in the lumber spine or lower extremities, such as the knee or ankle; (3) osteoarthritis (OA) of the knee with a Kellgren–Lawrence grade ≥ 2; or (4) dislocated or subluxated hip on the THA side evaluated as Crowe type 3 or 4 on plain radiograph.

**Figure 2:**
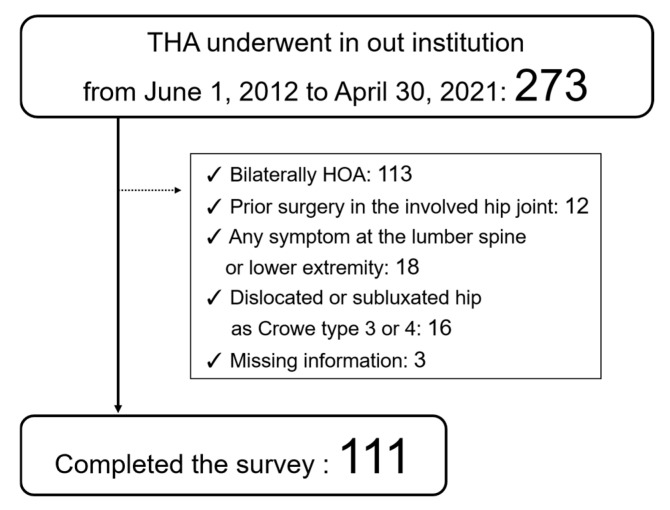
Participant flowchart.

Ultimately, 111 consecutive patients (83 females and 28 males) were included in the current study’s analyses. All patients completed the survey (Figure 2). All THAs were performed by five experienced orthopedic surgeons through an anterolateral spine approach [18,19,20].

The center of the acetabular components was placed close to the original hip center. The placement aimed for 40° radiographic inclination and 15° radiographic anteversion, as defined by Murray et al. [21], relative to the functional pelvic plane (FPP) [22]. This corrected the cerabrocaudal tilt in the sagittal plane, which contained the most anterior point of the pubic symphysis and bilateral superior iliac spines, forming the anterior pelvic plane in the supine position [21]. Acetabular components were placed using a mechanical guide [23] or CT-based navigation [24] with accuracies of approximately 3° and 2°, respectively [21,22]. A high hip center of <10 mm was deemed acceptable. The femoral component was positioned at an angle of 10–30° anteversion relative to the retrocondylar plane (RCP), which includes the bottom of the greater trochanter and bilateral femoral condyles [25]. Moreover, the combined anteversion, comprising the sum of the anteversion of the acetabular and femoral components, was adjusted to approximately 30–40° [26,27]. The leg length discrepancy was matched to the non-surgical healthy side, but the neck length was adjusted based on the soft tissue tension and risk of impingement or dislocation during surgery. Fluoroscopy was not used to confirm or adjust the offset and leg length discrepancies.

### 2.3. Measurement

A 3D bone model was reconstructed using the ZedView^®^ software [28,29,30] version 17.0.0 (Lexi, Tokyo, Japan) from CT images taken approximately 1 week after THA for preoperative planning and measuring implantation angle and position [24,31]. First, the 3D model of the pelvis was reconstructed and aligned to the anterior pelvic plane [21]. The acetabular offset was measured as the direction between the teardrop and the femoral head center parallel to the pelvis mediolateral axis (Figure 1a). Next, the 3D model of the femur was adjusted to the RCP, and the femoral offset was measured as the direction between the axis of the proximal femur and femoral head center parallel to the femoral mediolateral axis (Figure 1b). Subsequently, GFO was calculated according to the sum of the acetabular offset and femoral offset [32]. Therefore, the GFO in this study was unaffected by hip position (abduction/adduction and internal/external rotation), as AO and FO were measured in the same coordinate system before and after THA; alignment was retained among the participants [33].

**Figure 3:**
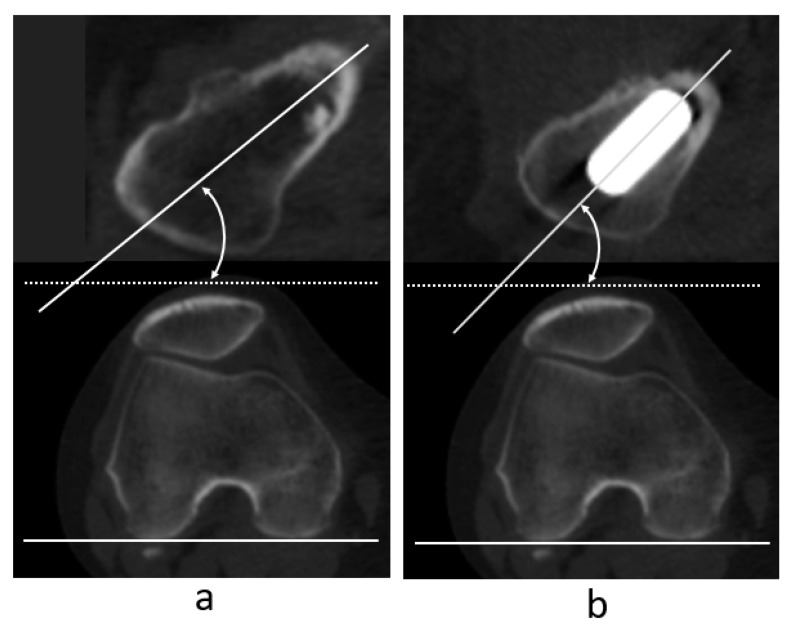
Definition of femoral anteversion (**a**) and stem anteversion (**b**).

For the femoral version (FV), femoral neck anteversion (FNA) was measured using the naive femur before THA and anteversion of the femoral component using the femur after implantation, with CT images aligned to the same femoral coordinate system. The femoral neck axis was defined as the midline between the anterior and posterior contour of the medullary space of the femoral neck in the axial plane just below the femoral head [34]. Femoral neck anteversion was defined as the angle between the femoral neck axis and the RCP (Figure 3a). Similarly, stem anteversion was defined as the angle between the stem axis and RCP in the same coordinate system as that used before surgery (Figure 3b). The difference between FNA and stem anteversion was defined as the change in FC (ΔFV).

**Figure 4:**
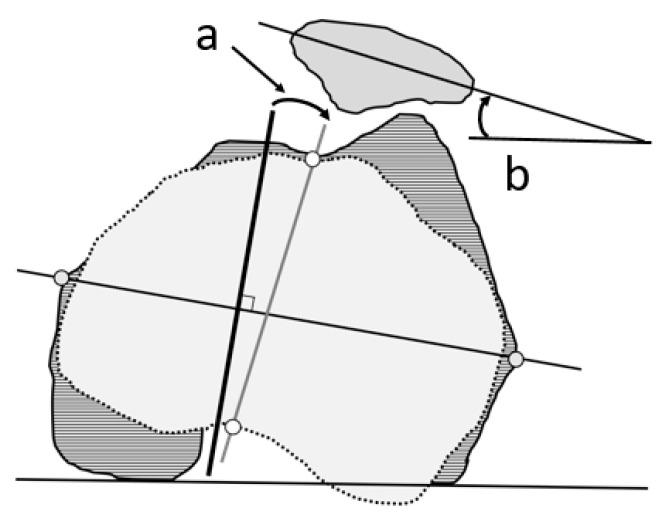
Definition of tibiofemoral rotation angle and patellar tilt angle. (a) Tibiofemoral rotation angle: angle between the line perpendicular to the transepicondylar axis and Akagi’s line. (b) Patellar tilt angle: angle between the transverse axis of the patella and the posterior cruciate in the axial plane of the femoral coordinates.

Regarding TFRA, the femoral anteroposterior (AP) axis was defined as the line perpendicular to the clinical epicondylar axis and the line connecting the medial and lateral epicondyles of the femur. Akagi’s line [35] was selected to represent the AP axis of the tibia. The TFRA was defined as the angle between the femoral and tibial AP axes in the axial plane of the femoral coordinate system (Figure 4). The PTA was defined as the angle between the transverse axis of the patella, the center of the patella, and the RCP in the axial plane of the femoral coordinate system (Figure 4). Negative values indicated internal rotation of the tibia and patella relative to the femur, while positive values indicated external rotation.

**Figure 5:**
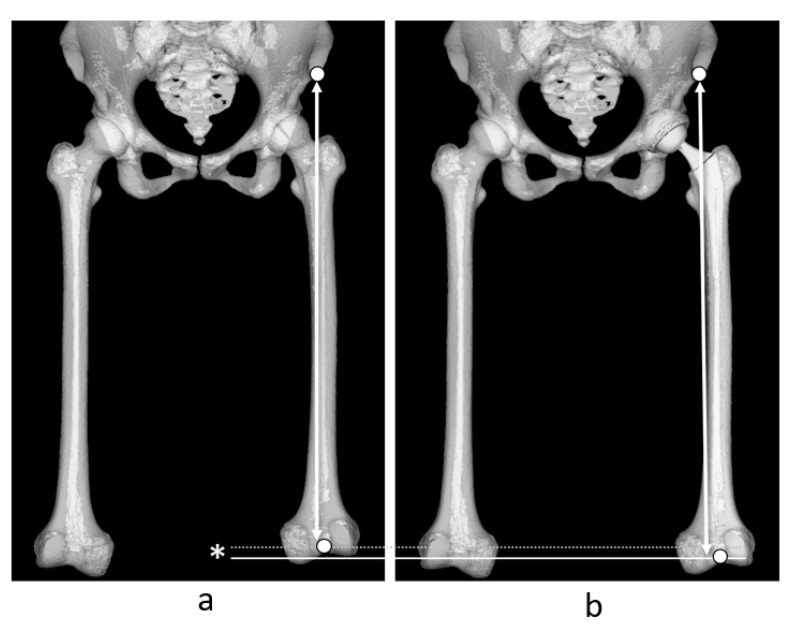
Definition of leg lengthening. Leg lengthening (*) was measured as the difference in the distance from the anterior superior iliac spine and the most distal point of the intercondylar fossa of the femur before (dotted line) (**a**) and after (solid line) (**b**) total hip arthroplasty.

Leg lengthening was determined by measuring the change in distance from the anterior superior iliac spine of the surgical site to the most caudal point of the intercondylar fossa of the femur before and after THA (Figure 5). Positive values indicated an increase in length after THA, while negative values indicated a decrease.

The differences between pre- and post-surgical GFO, FV, TFRA, and PTA were expressed as ΔGFO, ΔFV, ΔTFRA, and ΔPTA, respectively.

As body size adjustments, length measurements, i.e., GFO and leg-lengthening, were adjusted per 100 cm in height for easy calculation in actual 3D planning. The insertion angle of the acetabular component, radiographic inclination, and radiographic anteversion were expressed relative to preoperative FPP.

Experienced orthopedic surgeons evaluated hip function using mHHS within 2 months before surgery and 1 year after surgery.

### 2.4. Statistical Analyses

All data were analyzed using SPSS version 26 (SPSS Inc., Chicago, IL, USA). Linear regression was performed between the GFO, ΔFV, leg lengthening, ΔTFRA, and ΔPTA. Moreover, multiple logistic regression analysis was performed with ΔTFRA and ΔPTA as independent variables and weight, sex, body mass index (BMI), leg lengthening, and ΔFV as dependent variables. For correlation analysis, a post-hoc test was performed to evaluate statistical power (type II [β] error), with 0.5 as an effect size (d) and 0.05 as a type I (β) error. Intra- and inter-observer reliabilities were analyzed via the intraclass correlation coefficient (ICC). Intra-observer reliability was measured at least twice by one observer over intervals greater than 2 weeks. A *p* < 0.05 was considered statistically significant.

## 3. Results

The participants’ demographic data, including the implants, are presented in Table 1. The acetabular component was inserted at an inclination of 40.4 ± 4.8° and at an anteversion of 17.2 ± 6.2°. The mHHS score was significantly improved from 48.6 to 90.0.

The measured values for GFO, leg lengthening, FV, TFRA, and PTA before and after THA are presented in Table 2.

**Figure 6:**
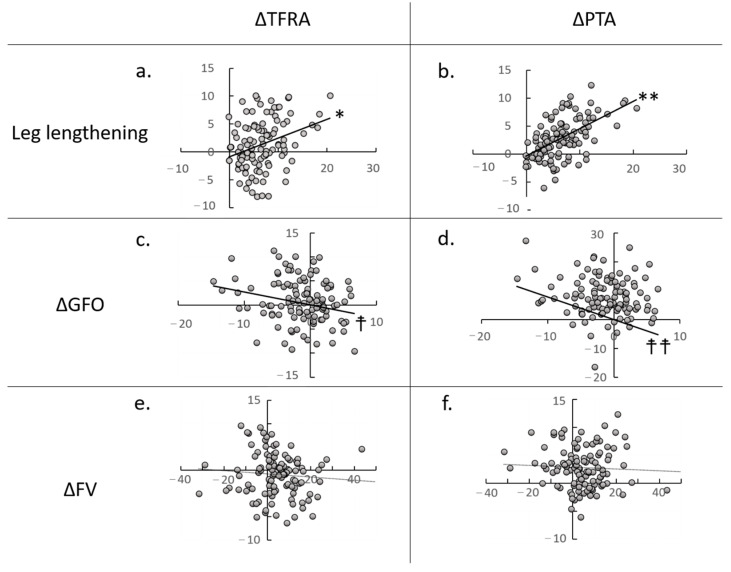
Correlation between leg lengthening (**a**,**b**), Δglobal femoral offset (GFO) (**c**,**d**), Δfemoral version (FV) (**e**,**f**), Δtibiofemoral rotation angle (TFRA) (**a**,**c**,**e**), and Δpatellar tilt angle (PTA) (**b**,**d**,**f**). Significant associations between leg lengthening and ΔTFRA (*) and ΔPTA (**). ΔGFO was significantly associated with ΔTFRA (☨) and ΔPTA(☨☨).

Leg lengthening was significantly positively correlated with TFRA and PTA, with greater leg lengthening correlated with greater TFRA (correlation coefficient: 0.317) and PTA (correlation coefficient: 0.566) external rotation of the tibia and patella relative to the femur (Figure 6, Table 3). However, ΔGFO was negatively correlated with ΔTFRA (correlation coefficient: 0.289) and ΔPTA (correlation coefficient: 0.342), with greater GFO leading to greater internal rotation of the tibia and patella relative to the femur. The effects of TFRA and PTA were stronger on leg lengthening than GFO. FVA was not associated with changes in TFRA or PTA (Figure 6, Table 3).

**Figure 7:**
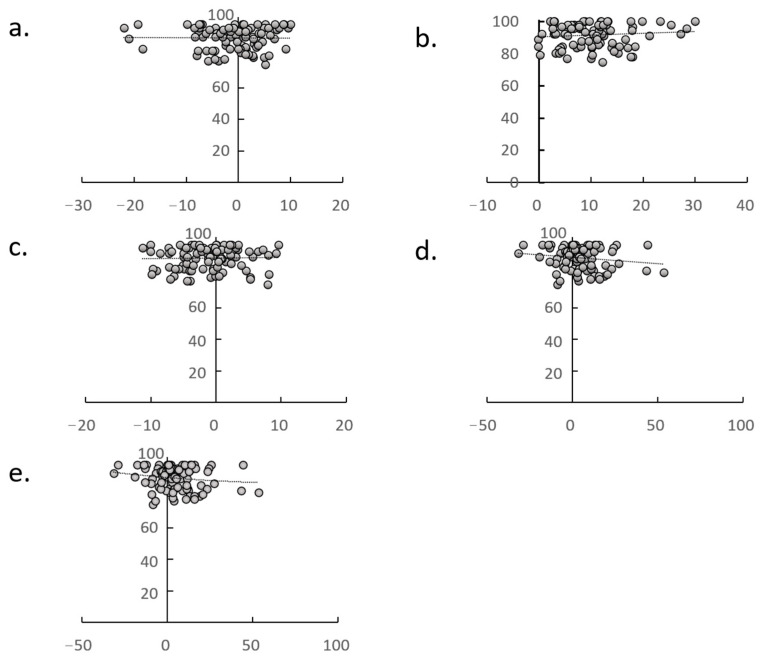
Correlation between m-Harris Hip Score (mHHS) and leg-lengthening, Δglobal femoral offset (GFO), Δtibiofemoral rotation angle (TFRA), Δpatellar tilt angle (PTA), and Δfemoral version (FV). No significant associations were observed between mHHS and (**a**) ΔGFO, (**b**) leg lengthening, (**c**) ΔTFRA, (**d**) ΔPTA, or (**e**) ΔFV.

ΔGFO, leg lengthening, ΔTFRA, and ΔPTA were all unrelated to mHHS at 1 year postoperatively (Figure 7). According to the multiple logistic regression analysis, ΔGFO and leg lengthening were the only factors that affected the ΔTFRA (correlation coefficient: 0.331, *p* = 0.020) and ΔPTA (correlation coefficient: 0.407, *p* = 0.004), respectively. Power analyses using correlations yielded power values of 0.950. The intra- and inter-observer reliabilities ranged from 0.823 to 0.909 for all measures. No adverse events that could affect the postoperative course, such as surgical site infections, dislocations, wound infections, or medical complications, were observed.

## 4. Discussion

The results of this study indicated that leg lengthening during THA correlated with TFRA and PTA, both of which tended to increase as leg lengthening increased. Moreover, ΔGFO and leg lengthening were the only factors impacting the ΔTFRA and ΔPTA, respectively. These results align with Kabasyashi et al. [36]. Leg lengthening likely increases iliotibial tract tension, leading to increased tension on the lateral side of the tibia and patella, causing them to rotate externally relative to the femur, thus increasing ΔTFRA and ΔPTA. This effect seemed more pronounced in PTA than in TFRA due to greater soft tissue support at the knee joint.

The TFRA and PT are reportedly 5–10° larger in patients with DDH than in healthy volunteers [15,16,17]. Although Yu et al. reported that the tibia externally rotates after THA [13], their measurement was not adjusted to a specific coordinate system, such as the RCP. In contrast, Iseki et al. reported that patellar dislocation occurs after THA due to increased patellar tilt and lateralization [37]. Moreover, Tokuhara et al. [14] found that if leg lengthening after THA exceeds 17 mm, anterior knee pain is common due to patellar lateralization. This supports the current study results.

In the current study, an increase in GFO tended to decrease TFRA and PTA; that is, the tibia and patella tended to rotate internally relative to the femur. This is the first report on the relationship between ΔGFO, ΔTFRA, and ΔPTA. Although the causes of these findings are unclear, an increase in the tension of the adductor muscles and medial soft tissue around the knee is likely a contributor, leading to increased tension of the tibia and patella in the direction of internal rotation. These changes are expected to reduce the strain on the patellofemoral joint and may improve knee pain. However, excessive GFO can cause hip pain and impair hip function [9,12], and knee OA increases with the medialization of the Mikulicz line [38]. Therefore, care should be taken to prevent the formation of excessive GFO.

In this study, ΔTFRA and ΔPTA were not associated with mHHS at 1 year postoperatively, although mHHS was optimal when the GFO on the THA side was approximately 5 mm larger than on the healthy side [1,9], and when the sum of the difference in GFO and leg length was equal to that on the healthy side [11,21]. Although ΔTFRA and ΔPTA are considered to have a lower priority than GFO and the sum of the difference in GFO and leg length, the results of this study are similar to those of “when the sum of the GFO difference and leg length difference is equal to that of the healthy side [11,12]”; that is, when a larger GFO than the healthy side results in a smaller leg lengthening.

Although many physicians likely avoid CT imaging postoperatively to limit radiation exposure, we believe CT imaging after THA is necessary to evaluate the precise implant angulation and the presence of postoperative fractures associated with THA. Indeed, GFO and leg lengthening evaluated on plain radiographs affected by abduction, adduction, and hip rotation are reportedly inaccurate [5,39]. Consequently, we considered it unsuitable to directly apply the values obtained from the study with plain radiographs in preoperative 3D planning, particularly for the anteversion of the femoral component, which affects GFO and leg lengthening. Therefore, our findings are considered valuable as the relevant factors could not be obtained through plain radiographs alone.

This study has certain limitations. First, only 111 patients were included, 96 of whom had DDH. This may limit the generalizability of the findings to other indications, such as osteonecrosis of the femoral head or primary HOA. Second, this study was conducted in a single center and was cross-sectional and retrospective. Third, the TFRA and PTA were evaluated by CT images preoperative and 1 week after THA. Although it is not possible to evaluate the alignment with matched coordinates unless the images are reconstructed from CT images, it is impractical to perform frequent examinations. Fourth, CT images were examined only in the supine position, limiting our evaluation. Fifth, as postoperative anterior knee pain was not assessed, the influence of TFRA and PTA remains unclear. Finally, the mHHS was limited to the 1st postoperative year, requiring additional long-term evaluation.

## 5. Conclusions

The results of this study indicate that leg lengthening during THA is related to TFRA and PT, both of which tend to increase with increased leg lengthening. Additionally, ΔGFO and leg lengthening were the only factors that impacted the ΔTFRA and ΔPTA, respectively. Accordingly, we caution against excessive leg lengthening as it may cause anterior knee pain. Setting the ΔGFO slightly larger than the healthy side might prevent this pain following THA. However, larger and more detailed studies are needed to evaluate the association between anterior knee pain and long-term outcomes after THA.

## Figures and Tables

**Table 1:** Participant details.

Sex (Female/Male)	28/83
Age (years) *	56.6 ± 10.4 (36–78)
Surgical side (right/left)	69/42
Primary disease	Developmental dysplasia of the hip: 96 Osteonecrosis of the femoral head: 14 Primary hip osteoarthritis: 2
Acetabular component	PlasmaFit^®^, B. Braun: 38 Trident^®^, Stryker: 73
Femoral component	BiCONTACT D stem^®^, B. Braun: 14 BiCONTACT E stem^®^, B. Braun: 24 Accolade II^®^, Stryker: 66 Exeter cemented stem^®^, Stryker: 7

* Mean ± standard deviation (range).

**Table 2:** Measurement value before and after total hip arthroplasty.

	Before Surgery	After Surgery	Difference: Δ
GFO (mm) *	66.7 ± 7.1 (51.8–87.1)	64.2 ± 7.0 (45.6–82.6)	−2.5 ± 6.7 (−22.0–9.9)
Leg lengthening (mm) *			10.0 ± 6.3 (−0.2–30.0)
FV (°)	23.4 ± 13.5 (−15.7–55.2)	26.9 ± 10.9 (−3.8–57.3)	3.5 ± 12.4(−31.6–53.6)
TFRA (°)	6.5 ± 7.4 (−11.3–26.2)	5.5 ± 7.0 (−15.8–20.7)	0.9 ± 4.6 (−9.6–11.3)
PTA (°)	12.8 ± 5.8 (−1.5–29.9)	15.5 ± 7.0 (−9.0–37.3)	2.7 ± 3.6 (−6.1–12.3)

* Mean ± standard deviation (range). Values were corrected to 100 cm of body height. GFO, global femoral offset; FV, femoral version; TFRA, tibiofemoral rotation angle; PTA, patellar tilt angle.

**Table 3:** Formulae calculated with regression equation.

	Formula	Correlation Coefficient	*p*-Value
*	y = 0.3355x − 0.8332	*r* = 0.317	0.008
**	y = 0.4868x − 0.3424	*r* = 0.566	<0.001
☨	y = −0.2712x	*r =* 0.289	0.018
☨☨	y = −0.7824x	*r =* 0.342	<0.001

(*), (**), (☨) and (☨☨) were regression formulae those were expressed in Figure 6a–d, respectively.

## Data Availability

The datasets generated and/or analyzed during the current study are available from the corresponding author upon reasonable request.

## Abbreviations

The following abbreviations are used in this manuscript:

AO	Acetabular offset
AP	Anteroposterior
CC	Correlation coefficient
CT	Computed tomography
DDH	Developmental dysplasia of the hip
FNA	Femoral neck anteversion
FO	Femoral offset
FPP	Functional pelvic plane
FV	Femoral version
GFO	Global femoral offset
HOA	Hip osteoarthritis
OA	Osteoarthritis
mHHS	m-Harris Hip Score
PTA	Patellar tilt angle
RCP	Retrocondylar plane
TFRA	Tibiofemoral rotation angle
THA	Total hip anthroplasty
